# External causes are leading causes of death in women of reproductive age: a registry study on maternal perinatal health, hypertensive pregnancy disorders and mortality in Finland

**DOI:** 10.1136/jech-2024-223438

**Published:** 2025-04-23

**Authors:** Tanja Saarela, Laura Peltomäki, Anna Kivioja, Tiina Jääskeläinen, Jari Haukka, Hannele Laivuori

**Affiliations:** 1 Tampere Center for Child, Adolescent, and Maternal Health Research, Faculty of Medicine and Health Technology, Tampere University, Tampere, Finland; 2 Department of Clinical Genetics, Kuopio University Hospital, Wellbeing Services County of North Savo, Kuopio, Finland; 3 Department of Obstetrics and Gynaecology, Satasairaala Central Hospital, Wellbeing Services County of Satakunta, Pori, Finland; 4 Department of Obstetrics and Gynaecology, University of Turku, Turku, Finland; 5 Department of Obstetrics and Gynaecology, Kanta-Häme Central Hospital, Wellbeing Services County of Kanta-Häme, Hameenlinna, Finland; 6 Medical and Clinical Genetics, University of Helsinki, Helsinki, Finland; 7 Department of Food and Nutrition, University of Helsinki, Helsinki, Finland; 8 Department of Public Health, Faculty of Medicine, University of Helsinki, Helsinki, Finland; 9 Institute for Molecular Medicine, Helsinki Institute of Life Science, University of Helsinki, Helsinki, Finland; 10 Department of Obstetrics and Gynaecology, Tampere University Hospital, Wellbeing Services County of Pirkanmaa, Tampere, Finland

**Keywords:** PREGNANCY, CARDIOVASCULAR DISEASES, MORTALITY, HYPERTENSION

## Abstract

**Background:**

There is a known association between pre-eclampsia (PE) and other hypertensive disorders of pregnancy (HDP), and increased risk of cardiovascular diseases (CVD). Reproductive history is associated with maternal mortality. We studied the causes of death in women of reproductive age and how a history of HDP affects mortality.

**Methods:**

We collected and analysed the perinatal data of national registers in a study of 555 345 women born in Finland during 1966—1990. The follow-up started from the woman’s first birth and ended on the first CVD, death or at the end of the follow-up of 23 years.

**Results:**

There were 295 373 women whose first birth was registered 1997—2019 and among them, 1287 deaths (cancer 493 deaths, preventable causes (suicide, accidents, alcohol, other external causes) 450 deaths, CVD 126 deaths). The diagnosis of PE or other HDP increased CVD mortality (risk ratio 2.69 (95% CI 1.40, 5.16) and 2.02 (95% CI 1.21, 3.38), respectively), compared with normotensive pregnancy. In the Poisson regression analyses, in women with other HDP than PE, a higher CVD mortality was found (mortality rate ratio 3.98, 95% CI 1.97, 8.04). Survival analysis showed reduced survival in women with PE for both CVD and all-cause mortality.

**Conclusions:**

Reproductive history, specifically preventable and CVD cause, has a significant role in mortality of women of reproductive age. Women have an increased risk of CVD death, and reduced survival of CVD mortality, if they have PE or other HDP, in the pregnancy associated with their first birth.

**WHAT IS ALREADY KNOWN ON THIS TOPIC**

**WHAT THIS STUDY ADDS**

- Reproductive history, specifically preventable and CVD causes, has a significant role in the mortality of women of reproductive age.
- A diagnosis of HDP is linked to reduced survival of CVD mortality.

**HOW THIS STUDY MIGHT AFFECT RESEARCH, PRACTICE OR POLICY**

- Pregnancy history offers an early opportunity for implementing preventive measures.

## Introduction 

Changes in maternal physiology during pregnancy increase the likelihood of complications, many of which have significant consequences on maternal health later in life. Among these, pre-eclampsia (PE) and its most severe complication eclampsia stand out as multisystem pregnancy disorders characterised by placental malperfusion and maternal vascular endothelial injury. They lead to hypertension, proteinuria and other organ dysfunction, whereas other hypertensive disorders of pregnancy (other HDP) refer to hypertension that arises during pregnancy without proteinuria or systemic complications. Further, the aetiology of PE and eclampsia is associated with placental dysfunction and a broader systemic response, whereas other HDP (such as gestational hypertension without proteinuria or pre-existing hypertension complicating pregnancy, childbirth and the puerperium) are typically driven by more localised blood-pressure-related disturbances without significant placental involvement. PE, along with other HDP, remains among the most common causes of maternal and fetal morbidity and mortality.^1^

The long-term health implications of HDP are profound. Women with a history of HDP, gestational diabetes mellitus, preterm birth, placental abruption and stillbirth face an elevated risk of developing cardiovascular diseases (CVDs) later in life.^2^ For example, low offspring birth weight has been linked to an increased risk of maternal atherosclerosis.^3^ After pregnancy, women with HDP are more likely to experience classic CVD risk factors, including chronic hypertension, renal dysfunction, dyslipidaemia, diabetes and subclinical atherosclerosis.^4^ Additionally, they tend to exhibit higher glucose, insulin, triglycerides and cholesterol levels, compared with women who had normotensive pregnancies.^5^ Notably, a history of HDP is associated with an average twofold increase in the risk of future cardiovascular events,^6^,^8^ which can manifest as early as the first decade following the affected pregnancies.^9 10^ However, it remains unclear whether this elevated risk is attributable to pre-existing CVD risk factors, the impact of HDP itself or a combination of both. The connection between HDP and CVD is further underscored by their shared risk factors,^11^ though the exact underlying pathophysiology linking the conditions is not yet fully understood.

CVD is the leading cause of mortality for women, yet significant sex-based disparities exist in both research and outcomes. In developed countries, the incidence of CVD has declined in men but not in women under 55 years of age.^12^ Furthermore, traditional metabolic risk factors for CVD appear to have a stronger impact on women more than men.^13^,^15^ When young women develop CVD, they have a disproportionately high risk of mortality compared with their male counterparts.^16^ Despite this, women, particularly those with pregnancy-related risk factors, have been under-represented in CVD clinical trials, limiting the ability to identify sex-specific and pregnancy-related characteristics of CVD.^17^ Recognising this gap, the American Heart Association has included PE, gestational diabetes mellitus and the birth of a growth-restricted child as pregnancy-related risk factors for CVD.^18^ These implications also carry higher medical costs due to increased healthcare utilisation and long-term adverse events, including mortality.^19^ Yet, despite this growing body of evidence, a history of HDP has not been incorporated into current CVD risk assessment tools.^20^

To address this gap, our study aimed to investigate the causes of death among women of reproductive age, with a particular focus on how perinatal history, PE and other HDP affect mortality, compared with women with normotensive pregnancies.

## Materials and methods

The study population was drawn from the national population register by the Digital and Population Data Services Agency and consisted of a 70% random sample of all girls born in Finland between 1966 and 1990 (N=555 345). For them, we collected all pregnancy and perinatal data of the Medical Birth Register from 1 January 1997 to 31 December 2019. The first date of study was selected because Finland adopted the codes from the 10th Revision of the International Classification of Diseases (ICD-10) in 1996, and our objective was to exclusively use these codes. The records were linked with The Care Register for Health Care (Hilmo, Finnish institute for health and welfare) and the national Cause of Death register (Statistics Finland) with personal identification numbers. All data were sent to The Finnish Social and Health Data Permit Authority Findata (FINDATA) for matching, combining and pseudonymisation. The data are stored, and the analyses were performed at Tampere University, through the remote access to the Fiona service (Statistics Finland), where the data are stored.

In the basic analysis of the study, pregnancy histories and outcomes of women with PE, and those of women with other HDP were analysed. They were compared with the history and outcomes of other women who have given birth. The following ICD-10 codes were used to determine PE: O11, O14.0, O14.1, O14.9, O15.0, O15.1, O15.2, O15.9 and other HDP: O10, O13, O16 and I10 (if set during pregnancy). The rest of the ICD-10 codes for pregnancy-related conditions, including O12.1 and O12.2, were included in the group of pregnancy without PE/HDP. The follow-up began at the first birth and continued until the first cardiac event or death, or until the end of the follow-up (31 December 2019). Accordingly, also previous pregnancies including miscarriages and induced abortions were reported. HPD in subsequent pregnancies was not considered. The diagnoses and deaths were studied until the age of 54 years, at its peak (1966—2019). The total mortality and cause-specific mortalities were studied. Only the main cause of death for each participant was used in the analysis. Mortality by causes of death was determined according to the classification of the national Cause of Death register with the following classes: cancer: 4—22; CVD: 27—30; alcohol 41; suicide 50; accidents 42—49; preventable: 41—50, 53.^21^

The data were modelled with Poisson regression, where death or cause of death (0/1) was used as the dependent variable, and length of follow-up as the offset term. Explanatory variables were age, year of birth and variables related to pregnancy and childbirth, and diagnoses before childbirth. Because our aim was to study the association between PE and the dependent variables, potential confounding variables, that is, variables affecting both the dependent variables and PE, were included in the models. Relationships between variables are presented in the causal diagram (Directed Acyclic Graph, DAG). Results are reported as mortality tables, Poisson regression results as mortality rate ratios (MRRs) and Kaplan-Meier curves. We used R software for all statistical analyses. The DAGs were drawn with the R package Dagitty.^22^

## Results

There were 295 373 women whose first childbirth was registered between 1997 and 2019. Associated with their first birth, 10 239 (3.5%) women had PE and 21 689 (7.3 %) had other HDP. Among them, women with PE were less likely to have a term pregnancy (≥37 weeks), compared with women with other HDP or women with normotensive pregnancies (73.3%, 92.4% and 94.7%, respectively, p<0.001). 44.5% of women with PE had a caesarean section during their first childbirth, whereas the proportion was 27.8% in women with other HDP and 19.0% in normotensive women (p<0.001). Child birth weight was significantly reduced in women with PE in the pregnancy leading to their first birth (p<0.001). The data description and baseline characteristics of the study population are represented in online supplemental table 1.

There were 1287 deaths among the study population (mortality rate per 100 000 person-years 35.6, 95% CI 33.7, 37.6), during the follow-up, at the age of less than 54 years. Mortality for all cancer contributed to 493 deaths (rate 140.1 (95% CI 128.2, 152.9), preventable causes together, excluding assaults, 450 deaths (rate 124.4, 95% CI 113.1, 136.4). Among preventable causes of death, suicide contributed to 196 deaths (rate 54.2, 95% CI 46.9, 62.3), accidents 159 deaths (rate 43.9, 95% CI 37.4, 51.3), alcohol 94 deaths (rate 26.0, 95% CI 21.0, 31.8) and other external causes and sequelae 1 death. CVD contributed to 126 deaths (rate 34.8, 95% CI 29.0, 41.5). The specific main causes of death are represented in online supplemental table 2.

Among deaths, 52 (4.0%) women had had PE in the first pregnancy leading to their first birth and 102 (7.9%) had other HDP. The unadjusted mortality tables for all-cause, CVD and preventable cause mortality are represented in tables1^3^, respectively. Having any HDP diagnosis increased the risk of CVD death (risk ratio (RR) 2.69, 95% CI 1.40, 5.16 for PE and 2.02, 95% CI 1.21, 3.38, for other HDP), compared with having a normotensive pregnancy, but the difference was not significant in preventable causes or all-cause mortality. Preterm birth was associated with a higher all-cause mortality (RR 1.72, 95% CI 1.39, 2.13 for 34—36+6 weeks and 2.14, 95% CI 1.61, 2.86 for <34 weeks). Of other variables, a psychiatric diagnosis, smoking during pregnancy and having to undergo caesarean section as the mode of delivery increased the mortality of all causes, of CVD and of preventable causes. The RRs for mortality of preventable causes and of all-causes, in women with miscarriages and induced abortions before their first birth, were increased (2.63, 95% CI 1.98, 3.49 and 1.91, 95% CI 1.58, 2.31, respectively), compared with other women. Having body mass index (BMI) ≥35 kg/m^2^ was associated with higher all-cause and CVD mortality (RR 1.66, 95% CI 1.10, 2.51 and RR 4.64, 95% CI 1.97, 11.4), respectively). There was no significant association between PE or other HDP and cancer mortality (RR 0.44, 95% CI 0.14, 1.36 for PE and RR 0.43, 95% CI 0.16, 1.15 for other HDP). Daily smoking after the first trimester was associated with higher mortality of cancer (RR 1.33, 95% CI 1.12, 1.57).

**Table 1:** All-cause mortality (per 100 000 person-years) for Statistics Finland classification causes of death^21^

Variable	Group	Person-years	Event	Rate (95% **CI**)	RR (95% **CI**)
Hypertension status during pregnancy	Pre-eclampsia	1.21	52	42.97 (32.09 to 56.35)	1.22 (0.93 to 1.61)
	Other hypertensive pregnancy	2.74	102	37.22 (30.35 to 45.18)	1.06 (0.87 to 1.30)
	No hypertension	32.23	1133	35.15 (33.13 to 37.26)	ref.
Psychiatric diagnosis (ICD-10)	No	36.12	1272	35.22 (33.31 to 37.21)	ref.
	Yes	0.07	15	225.66 (126.30 to 372.19)	6.41 (3.85 to 10.66)
Pathological OGTT	No	34.38	1229	35.75 (33.78 to 37.80)	ref.
	Yes	1.80	58	32.21 (24.46 to 41.64)	0.90 (0.69 to 1.17)
Previous pregnancies	No previous pregnancy	34.39	1171	34.05 (32.13 to 36.06)	ref.
	≥1	1.79	116	64.99 (53.71 to 77.95)	1.91 (1.58 to 2.31)
Woman’s birthyear	1966–1969	4.63	274	59.23 (52.42 to 66.67)	ref.
	1970–1979	19.76	684	34.61 (32.06 to 37.20)	0.58 (0.51 to 0.67)
	1980–1990	11.79	329	27.90 (24.97 to 31.08)	0.47 (0.40 to 0.55)
Age (years)	13–20	1.34	74	55.10 (43.27 to 69.18)	ref.
	20–25	8.54	321	37.57 (33.57 to 41.91)	0.68 (0.53 to 0.88)
	25–30	14.13	406	28.73 (26.00 to 31.66)	0.52 (0.41 to 0.67)
	30–35	9.60	361	37.60 (33.82 to 41.69)	0.68 (0.53 to 0.88)
	≥35	2.56	125	48.80 (40.62 to 58.14)	0.89 (0.66 to 1.18)
Mode of delivery	Vaginal	29.10	961	33.03 (30.97 to 35.19)	ref.
	Caesarean section	7.09	326	46.01 (41.15 to 51.28)	1.39 (1.23 to 1.58)
Smoking during pregnancy	No	29.99	893	29.78 (27.86 to 31.80)	ref.
	Yes	6.20	394	63.58 (57.46 to 70.18)	2.14 (1.90 to 2.40)
BMI (kg/m^2^)	0–30	15.43	394	25.53 (23.07 to 28.18)	ref.
	30–35	1.21	30	24.75 (16.70 to 35.33)	0.97 (0.67 to 1.41)
	≥35	0.57	24	42.29 (27.22 to 63.22)	1.66 (1.10 to 2.51)
Duration of pregnancy (weeks)	≥37^+0^	33.85	1141	33.70 (31.78 to 35.72)	ref.
	34^+0^–36^+6^	1.57	91	57.99 (46.69 to 71.20)	1.72 (1.39 to 2.13)
	<34^+0^	0.66	48	72.28 (53.29 to 95.83)	2.14 (1.61 to 2.86)

Previous pregnancies include miscarriages and induced abortions before the first birth.

BMI, body mass index; ICD-10, 10th Revision of the International Classification of Diseases; OGTT, oral glucose tolerance test; ref, reference; RR, risk ratio.

**Table 2:** Cardiovascular disease mortality (per 100 000 person-years) for Statistics Finland classification causes of death^21^

Variable	Group	Person years	Event	Rate (95% CI)	RR (95% CI)
Hypertension status during pregnancy	Pre-eclampsia	1.21	10	8.26 (3.96 to 15.20)	2.69 (1.40 to 5.16)
	Other hypertensive pregnancy	2.74	17	6.20 (3.61 to 9.93)	2.02 (1.21 to 3.38)
	No hypertension	32.23	99	3.07 (2.50 to 3.74)	ref.
Psychiatric diagnosis (ICD-10)	No	36.12	124	3.43 (2.86 to 4.09)	ref.
	Yes	0.07	2	30.09 (3.64 to 108.69)	8.76 (2.17 to 35.43)
Pathological OGTT	No	34.38	119	3.46 (2.86 to 4.14)	ref.
	Yes	1.8	7	3.89 (1.56 to 8.01)	1.12 (0.52 to 2.41)
Previous pregnancies	No	34.39	116	3.37 (2.79 to 4.05)	ref.
	≥1	1.79	10	5.60 (2.69 to 10.30)	1.66 (0.87 to 3.17)
Woman’s birthyear	1966–1969	4.63	34	7.35 (5.09 to 10.27)	ref.
	1970–1979	19.76	78	3.95 (3.12 to 4.93)	0.54 (0.36 to 0.80)
	1980–1990	11.79	14	1.19 (0.65 to 1.99)	0.16 (0.09 to 0.30)
Age (years)	13–20	1.34	6	4.47 (1.64 to 9.72)	ref.
	20–25	8.54	24	2.81 (1.80 to 4.18)	0.63 (0.26 to 1.54)
	25–30	14.13	38	2.69 (1.90 to 3.69)	0.60 (0.25 to 1.42)
	30–35	9.6	39	4.06 (2.89 to 5.55)	0.91 (0.39 to 2.15)
	≥35	2.56	19	7.42 (4.47 to 11.58)	1.66 (0.66 to 4.16)
Mode of delivery	Vaginal	29.1	88	3.03 (2.43 to 3.73)	ref.
	Caesarean section	7.09	38	5.36 (3.80 to 7.36)	1.77 (1.21 to 2.59)
Smoking during pregnancy	No	29.99	93	3.10 (2.50 to 3.80)	ref.
	Yes	6.2	33	5.33 (3.67 to 7.48)	1.72 (1.15 to 2.55)
BMI (kg/m2)	0–30	15.43	35	2.27 (1.58 to 3.15)	ref.
	30–35	1.21	85	2.48 (0.51 to 7.23)	1.09 (0.34 to 3.55)
	≥35	0.57	6	10.62 (3.90 to 23.12)	4.64 (1.97 to 11.14)
Duration of pregnancy (weeks)	≥37^+0^	33.85	106	3.13 (2.56 to 3.79)	ref.
	34^+0^–36^+6^	1.57	16	10.20 (5.83 to 16.56)	3.26 (1.93 to 5.51)
	<34^+0^	0.66	4	6.02 (1.64 to 15.42)	1.92 (0.71 to 5.22)

Previous pregnancies include miscarriages and induced abortions before the first birth.

BMI, body mass index; ICD-10, 10th Revision of the International Classification of Diseases; OGTT, oral glucose tolerance test; ref, reference; RR, risk ratio.

**Table 3:** Preventable cause (suicide, accidents, alcohol, poisoning) mortality per 100 000 person-years, for Statistics Finland classification causes of death^21^

Variable	Group	Person-years	Event	Rate (95% **CI**)	RR (95% **CI**)
Hypertension status during pregnancy	Pre-eclampsia	1.21	14	11.57 (6.33 to 19.41)	0.92 (0.54 to 1.57)
	Other hypertensive pregnancy	2.74	32	11.67 (7.99 to 16.48)	0.93 (0.65 to 1.34)
	No hypertension	32.23	404	12.53 (11.34 to 13.81)	ref.
Psychiatric diagnosis (ICD-10)	No	36.12	441	12.21 (11.19 to 13.41)	ref.
	Yes	0.07	9	135.40 (61.91 to 257.02)	11.09 (5.73 to 21.45)
Pathological OGTT	No	34.382	435	12.65 (11.49 to 13.90)	ref.
	Yes	1.801	15	8.33 (4.66 to 13.74)	0.66 (0.39 to 1.10)
Previous pregnancies	No previous pregnancy	34.391	396	11.51 (10.41 to 12.71)	ref.
	≥1	1.785	54	30.26 (22.73 to 39.48)	2.63 (1.98 to 3.49)
Woman’s birthyear	1966–1969	4.626	81	17.51 (13.91 to 21.76)	ref.
	1970–1979	19.76	212	10.73 (9.33 to 12.27)	0.61 (0.47 to 0.79)
	1980–1990	11.79	157	13.31 (11.31 to 15.57)	0.76 (0.58 to 0.99)
Age (years)	13–20	1.34	51	37.98 (28.28 to 49.93)	ref.
	20–25	8.54	153	17.91 (15.18 to 20.98)	0.47 (0.34 to 0.65)
	25–30	14.13	118	8.35 (6.91 to 10.00)	0.22 (0.16 to 0.31)
	30–35	9.60	104	10.83 (8.85 to 13.13)	0.29 (0.20 to 0.40)
	≥35	2.56	24	9.37 (6.00 to 13.94)	0.25 (0.15 to 0.40)
Mode of delivery	Vaginal	29.10	340	11.69 (10.48 to 13.00)	ref.
	Caesarean section	7.07	110	15.52 (12.76 to 18.71)	1.33 (1.07 to 1.65)
Smoking during pregnancy	No	29.99	246	8.20 (7.21 to 9.30)	ref.
	Yes	6.20	204	32.92 (28.56 to 37.76)	4.01 (3.33 to 4.83)
BMI (kg/m2)	0–30	15.43	120	7.76 (6.45 to 9.30)	ref.
	30–35	1.21	10	8.25 (3.96 to 15.17)	1.06 (0.56 to 2.02)
	≥35	0.57	7	12.39 (4.98 to 25.53)	1.59 (0.74 to 3.41)
Duration of pregnancy (weeks)	≥37^+0^				ref.
	<37^+0^	2.23	33	14.78 (10.17 to 20.75)	1.21 (0.85 to 1.73)

Previous pregnancies include miscarriages and induced abortions before the first birth.

BMI, body mass index; ICD-10, 10th Revision of the International Classification of Diseases; OGTT, oral glucose tolerance test; ref, reference; RR, risk ratio.

According to the Poisson regression analyses (table 4), PE was not associated with all-cause mortality rate, but in women with other HDPs, for CVD cause of death, a higher risk was shown, compared with women with a normotensive pregnancy (MRR 3.98, 95% CI 1.97, 8.04).

**Table 4:** MRRs based on Poisson regression models

	All causes N=1287	CVD N=126	Cancer N=493	Alcohol N=94	Suicide N=196	Accidents N=159	Preventable N=450
	MRR (95% CI)	MRR (95% CI)	MRR (95% CI)	MRR (95% CI)	MRR (95% CI)	MRR (95% CI)	MRR (95% CI)
No hypertension (reference)							
Pre-eclampsia	1.10 (0.70 to 1.74)	2.05 (0.67 to 6.24)	0.46 (0.17 to 1.25)	1.41 (0.17 to 11.98)	0.85 (0.20 to 3.58)	2.21 (0.67 to 7.30)	1.36 (0.59 to 3.15)
Other hypertensive pregnancy	1.16 (0.84 to 1.60)	3.98 (1.97 to 8.04)	0.53 (0.27 to 1.05)	1.32 (0.28 to 6.19)	0.54 (0.17 to 1.74)	2.01 (0.88 to 4.55)	1.16 (0.63 to 2.11)

Mortality rate ratios (MRR for pre-eclampsia, based on Poisson regression models reporting 95% CIs and adjusted for confounding variables. The included confounders were age, calendar period, smoking during pregnancy, body mass index, pathological oral glucose tolerance test, mode of delivery, length of gestation, preterm delivery <37 weeksks and F-diagnosis (mental and behavioural disorders classified in ICD-10 (international classification of diseases 10th revision) to F00–F99).

CVD, cardiovascular diseases; MRR, mortality rate ratio; Preventable, preventable causes of death, including suicide, accidents, alcohol and poisoning.

37 weeks

**Figure 1:**
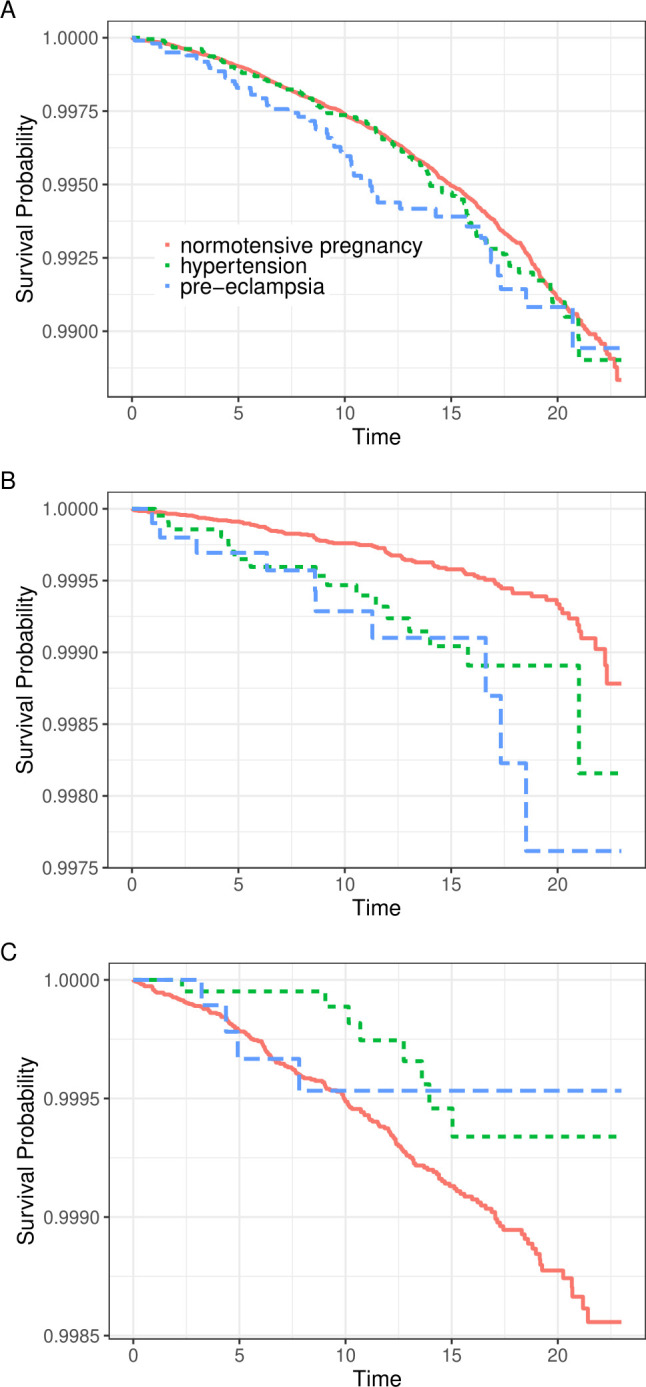
The survival curves for (A) all-cause mortality, (B) cardiovascular diseases mortality and (C) suicide mortality, in women with pre-eclampsia (‘pre-eclampsia’), other hypertensive disorders of pregnancy (‘hypertension’) and normotensive pregnancy.

The survival curves for all-cause mortality (A), CVD mortality (B) and suicide mortality (C), in PE, other HDP and normotensive pregnancy groups are shown in figure 1. For CVD mortality, the reduced survival among women who had PE or other HDP, in the pregnancy associated with their first birth, was consistent throughout the follow-up. For all-cause mortality, a reduced survival among women with PE, but not with other HDP, was seen. Survival of suicide mortality seemed increased in women with other HDP, but because of a limited number of participants in this group, CI remained wide.

The associated DAGs are shown in online supplemental figure 1.

## Discussion

In women of reproductive age, after cancer, the external preventable causes (suicides, alcohol, accidents and poisoning) were the next common cause of death, then followed by CVD. Women with PE and other HDP in the pregnancy leading to their first birth were both over-represented in the group of women who died of a CVD cause, but not in women who died of a preventable cause or of cancer, when comparing to women with normotensive pregnancies. Both PE and HDP lead to reduced survival in CVD mortality, and PE to reduced survival also in all-cause mortality. However, in Poisson regression analysis, not PE but specifically other HDP predicted a higher risk of CVD death, compared with women with a normotensive first pregnancy. Similarly, the baseline characteristics of the two disease groups were somewhat different: women with PE in the pregnancy associated with their first birth were less likely to be severely overweight and they more likely had a multiple pregnancy, preterm birth or urgent caesarean section, compared with all women and to women with other HDP (online supplemental table 1). The differences between the groups may reflect long-term complications of other CVD risk factors or effects of environmental and social conditions in other HDP, whereas PE may confer a distinct complex immunological and genetic aetiology.

Our findings emphasise the importance of including young women in future prevention programmes in healthcare, already during their pregnancy and following the first years after the pregnancy. Previous literature on CVD after any HDP has been consistent with the finding of increased morbidity, but in some of the publications, reviewed by Ahmed *et al*, the effect of PE or other HDP on CVD mortality has been modest.^23^ In a Norwegian population cohort study, covering 42 years’ follow-up, women with PE in the first pregnancy had a 1.9-fold CVD mortality rate, compared with women with no PE, but no increase in other causes of death.^24^ Another Norwegian study with a 13 years’ follow-up showed a 2.7-fold mortality in women who had a PE and preterm birth, when compared with women with no PE and term birth. Also, for cardiovascular causes, an increased mortality was shown. No increase in all-cause mortality was seen in women who had PE but whose pregnancy reached full term.^25^ However, there are some previous reports on increased all-cause mortality in women with HDP, compared with women who remain normotensive in their pregnancy.^20 26^

In our study, having a psychiatric diagnosis was associated with increased mortality rates of all causes, preventable causes and of CVD causes (table 1). In a study of a previous birth cohort from Finland and Denmark, patients with mental disorders had a higher mortality than the general population, and there was an excess in mortality in women at ages under 50 years, compared with general population.^27^ Lega *et al* studied women who died in Italy by suicide during pregnancy or within 1 year after giving birth, induced abortion or miscarriage. Suicides represented 12% of the maternal deaths, and 40% of the suicides happened without previous psychiatric history.^28^ Other studies on short-term mortality suggest that giving birth may act as a protective factor, as mortality and suicide rates appear to be higher following miscarriage or abortion compared with childbirth.^29^,^31^ Accordingly, we found an association between a history of a previous pregnancy before the first birth (miscarriage or induced abortion) and increased mortality, especially mortality of preventable causes. This may be associated with complex psychological and social implications of perinatal history that need further exploration. Further study is needed on the possible mechanisms and risk factors that fell outside the primary scope of this study.

Strengths of the study were its use of a large national registry data of women of reproductive age, including detailed perinatal history and outcome, as well as data on risk factors including BMI and smoking. The Finnish Medical Birth Registry and the other registers that were used are considered good in quality, and their accuracy has been investigated in previous validation studies.^32^,^35^

A limitation of the study was that, although the follow-up period extended up to 23 years, the oldest participants were only 54 years old. Consequently, due to the low number of deaths among young women in Finland, the study lacked sufficient power to analyse cause-specific mortality across different HDP groups. Furthermore, the association between reproductive history and mortality may be different later in women’s lives. It is possible that a few of the older participants would have had a CVD prior to the start of this data collection, or that some women who emigrated from Finland were not counted. However, as women with a history of HDP were compared with those with normotensive pregnancies, any errors related to pre-existing CVD would likely balance out when comparing the groups.

Our results support the previous finding that reproductive history seems to influence postreproductive mortality. Especially preventable and CVD causes have a significant role in the mortality of women of reproductive age. How the recurrence of HDP in following pregnancies, or the diagnosis of preterm birth, early CVD or a psychiatric condition, affects morbidity and mortality in women of reproductive age still needs further research.

## Supplementary material

10.1136/jech-2024-223438 online supplemental figure 1

10.1136/jech-2024-223438 online supplemental table 1

10.1136/jech-2024-223438 online supplemental table 2

## Data Availability

Data may be obtained from a third party and are not publicly available.
