# Supplementary figures and images for: External causes are leading causes of death in women of reproductive age: a registry study on maternal perinatal health, hypertensive pregnancy disorders and mortality in Finland
Source: J Epidemiol Community Health. 2025 Apr 23;79(9):e223438. doi: 10.1136/jech-2024-223438 (PMC12418550; doi:10.1136/jech-2024-223438)

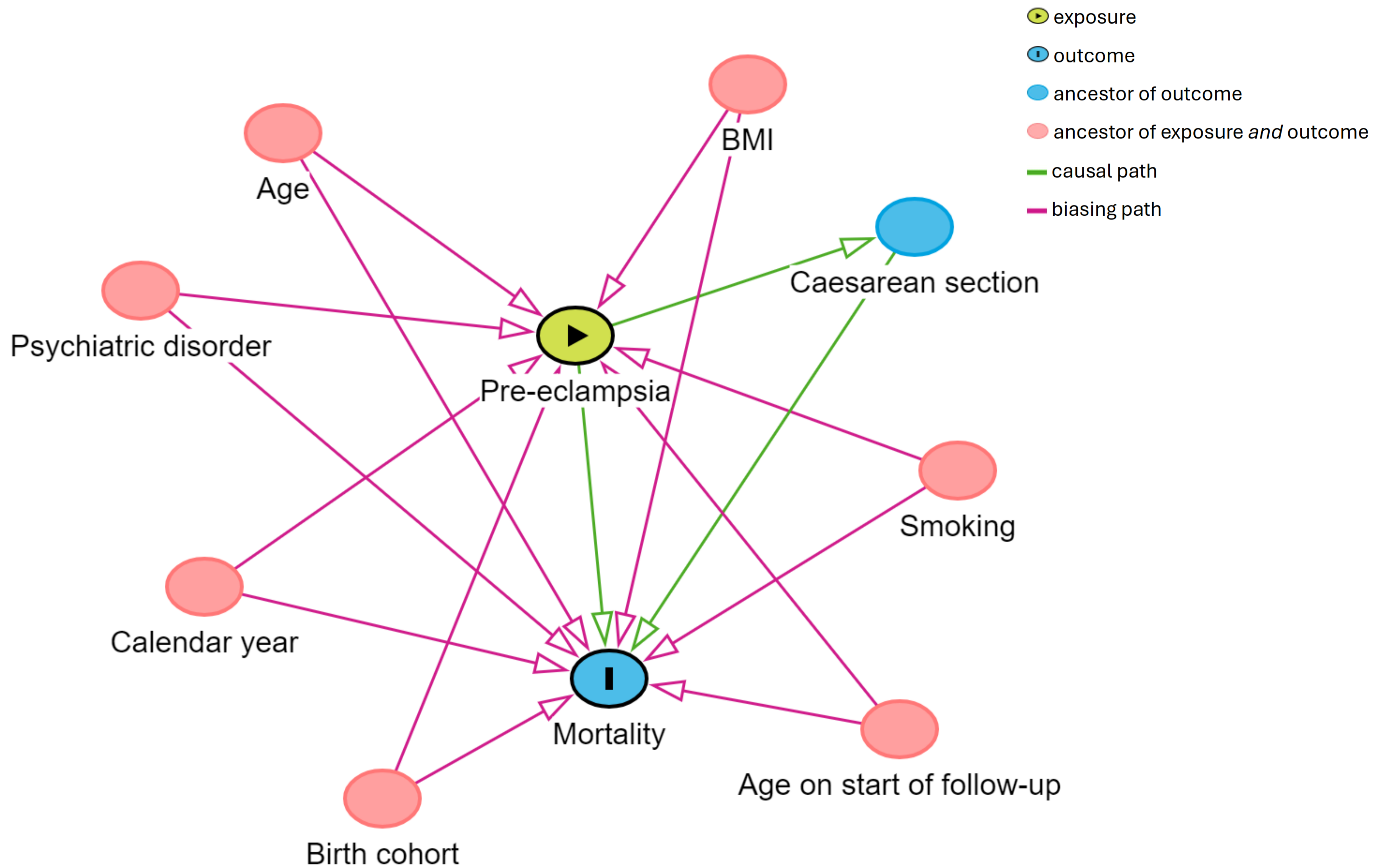

Supplement: online supplemental figure 1 [file jech-79-9-s001.pdf]
