# Supplementary material for: External causes are leading causes of death in women of reproductive age: a registry study on maternal perinatal health, hypertensive pregnancy disorders and mortality in Finland
Source: J Epidemiol Community Health. 2025 Apr 23;79(9):e223438. doi: 10.1136/jech-2024-223438 (PMC12418550; doi:10.1136/jech-2024-223438)
Supplement: online supplemental table 1 [file jech-79-9-s002.docx]

|  | ***Overall*** | ***Pre-eclampsia*** | ***Other hypertensive pregnancy*** | ***No hypertension*** | ***p*** | ***test*** | ***Missing*** |
| --- | --- | --- | --- | --- | --- | --- | --- |
| **N** | 295373 | 10239 | 21689 | 263445 |  |  |  |
| **Year of birth (%)** |  |  |  |  | <0.001 |  | 0.0 |
| **1966–1969** | 24727 (8.4) | 861 (8.4) | 2448 (11.3) | 21418 (8.1) |  |  |  |
| **1970–1979** | 130690 (44.2) | 4653 (45.4) | 10610 (48.9) | 115427 (43.8) |  |  |  |
| **1980–1990** | 139956 (47.4) | 4725 (46.1) | 8631 (39.8) | 126600 (48.1) |  |  |  |
| **Age (years) (%)** |  |  |  |  | <0.001 |  | 0.0 |
| **13–20** | 8036 (2.7) | 226 (2.2) | 428 (2.0) | 7382 (2.8) |  |  |  |
| **20–25** | 56940 (19.3) | 1865 (18.2) | 3623 (16.7) | 51452 (19.5) |  |  |  |
| **25–30** | 111103 (37.6) | 3675 (35.9) | 7754 (35.8) | 99674 (37.8) |  |  |  |
| **30–35** | 88068 (29.8) | 3070 (30.0) | 6595 (30.4) | 78403 (29.8) |  |  |  |
| **> 35** | 31226 (10.6) | 1403 (13.7) | 3289 (15.2) | 26534 (10.1) |  |  |  |
| **Weight (kg) (median [IQR])** | 64.0 [57.0, 73.0] | 66.0 [59.0, 78.0] | 70.0 [61.0, 83.0] | 63.0 [57.0, 72.0] | <0.001 | nonnorm | 34.4 |
| **Height (cm) (median [IQR])** | 166.0 [162.0, 170.0] | 165.0 [161.0, 170.0] | 166.00 [162.0, 170.0] | 166.0 [162.0, 170.0] | <0.001 | nonnorm | 32.3 |
| **BMI (kg/m^2^) (%)** |  |  |  |  | <0.001 |  | 34.4 |
| **0–30** | 172341 (89.0) | 5716 (81.1) | 10295 (74.5) | 156330 (90.5) |  |  |  |
| **30–35** | 14378 (7.4) | 844 (12.0) | 2068 (15.0) | 11466 (6.6) |  |  |  |
| **> 35** | 6958 (3.6) | 490 (7.0) | 1450 (10.5) | 5018 (2.9) |  |  |  |
| **Smoking status (%)** |  |  |  |  | <0.001 |  | 0.0 |
| **No** | 240827 (81.5) | 8584 (83.8) | 17930 (82.7) | 214313 (81.4) |  |  |  |
| **Quitted smoking during the 1. trimester** | 16892 (5.7) | 583 (5.7) | 1367 (6.3) | 14942 (5.7) |  |  |  |
| **Smoked after 1. trimester daily** | 30991 (10.5) | 822 (8.0) | 1933 (8.9) | 28236 (10.7) |  |  |  |
| **Smoked after 1. trimester occasionally** | 179 (0.1) | 4 (0.0) | 11 (0.1) | 164 (0.1) |  |  |  |
| **No information** | 6484 (2.2) | 246 (2.4) | 448 (2.1) | 5790 (2.2) |  |  |  |
| **F-diagnosis (ICD-10) (%)** | 972 (0.3) | 54 (0.5) | 80 (0.4) | 838 (0.3) | 0.001 |  | 0.0 |
| **Number of fetuses (%)** |  |  |  |  | <0.001 |  | 0.0 |
| **1** | 290608 (98.4) | 9527 (93.0) | 21164 (97.6) | 259917 (98.7) |  |  |  |
| **2** | 4697 (1.6) | 707 (6.9) | 519 (2.4) | 3471 (1.3) |  |  |  |
| **3** | 67 (0.0) | 5 (0.0) | 6 (0.0) | 56 (0.0) |  |  |  |
| **Previous pregnancies (>1) (%)** | 15865 (5.4) | 581 (5.7) | 1222 (5.6) | 14062 (5.3) | 0.068 |  | 0.0 |
| **Positive OGTT (%)** | 24269 (8.2) | 1250 (12.2) | 2761 (12.7) | 20258 (7.7) | <0.001 |  | 0.0 |
| **Length of gestation (weeks) (%)** |  |  |  |  | <0.001 |  | 0.2 |
| **< 28^+0^** | 1027 (0.3) | 147 (1.4) | 61 (0.3) | 819 (0.3) |  |  |  |
| **28^+0^-31^+6^** | 1757 (0.6) | 416 (4.1) | 159 (0.7) | 1182 (0.4) |  |  |  |
| **32^+0^-33^+6^** | 2409 (0.8) | 452 (4.4) | 206 (1.0) | 1751 (0.7) |  |  |  |
| **34^+0^-36^+6^** | 13087 (4.4) | 1708 (16.7) | 1222 (5.6) | 10157 (3.9) |  |  |  |
| **37^+0^-42^+0^** | 265397 (90.0) | 7424 (72.6) | 19477 (90.0) | 238496 (90.7) |  |  |  |
| **>42^+0^** | 11077 (3.8) | 73 (0.7) | 526 (2.4) | 10478 (4.0) |  |  |  |
| **Mode of delivery (%)** |  |  |  |  | <0.001 |  | 0.0 |
| **Vaginal** | 189892 (64.3) | 4431 (43.3) | 12419 (57.3) | 173042 (65.7) |  |  |  |
| **Breech, vaginal birth** | 2386 (0.8) | 45 (0.4) | 89 (0.4) | 2252 (0.9) |  |  |  |
| **Forceps** | 357 (0.1) | 8 (0.1) | 33 (0.2) | 316 (0.1) |  |  |  |
| **Vacuum** | 41915 (14.2) | 1197 (11.7) | 3108 (14.3) | 37610 (14.3) |  |  |  |
| **Planned C-section** | 17668 (6.0) | 894 (8.7) | 1449 (6.7) | 15325 (5.8) |  |  |  |
| **Urgent C-section** | 27890 (9.4) | 2541 (24.8) | 3004 (13.9) | 22345 (8.5) |  |  |  |
| **Emergency C-section** | 3230 (1.1) | 174 (1.7) | 285 (1.3) | 2771 (1.1) |  |  |  |
| **C-section, not planned (before 2004)** | 11921 (4.0) | 946 (9.2) | 1296 (6.0) | 9679 (3.7) |  |  |  |
| **No information** | 101 (0.0) | 3 (0.0) | 6 (0.0) | 92 (0.0) |  |  |  |
| **Birthweight (g) (mean (SD))** | 3420.01 (559.88) | 2959.50 (801.16) | 3327.60 (614.14) | 3445.52 (534.66) | <0.001 |  | 0.0 |
| **Birthheight (cm) (mean (SD))** | 49.89 (2.63) | 47.86 (4.12) | 49.58 (2.87) | 49.99 (2.50) | <0.001 |  | 0.7 |
| **Head circumference (cm) (mean (SD))** | 34.72 (1.79) | 33.70 (2.69) | 34.57 (1.91) | 34.77 (1.72) | <0.001 |  | 34.2 |
| **APGAR at 1min (mean (SD))** | 8.42 (1.40) | 8.05 (1.73) | 8.28 (1.54) | 8.45 (1.37) | <0.001 |  | 0.2 |
| **APGAR at 5 min (%)** |  |  |  |  | <0.001 |  | 41.0 |
| **0** | 588 (0.3) | 22 (0.3) | 57 (0.4) | 509 (0.3) |  |  |  |
| **1** | 137 (0.1) | 10 (0.2) | 12 (0.1) | 115 (0.1) |  |  |  |
| **2** | 191 (0.1) | 21 (0.3) | 14 (0.1) | 156 (0.1) |  |  |  |
| **3** | 343 (0.2) | 40 (0.6) | 34 (0.3) | 269 (0.2) |  |  |  |
| **4** | 578 (0.3) | 60 (0.9) | 52 (0.4) | 466 (0.3) |  |  |  |
| **5** | 959 (0.6) | 88 (1.4) | 100 (0.8) | 771 (0.5) |  |  |  |
| **6** | 2986 (1.7) | 214 (3.3) | 305 (2.4) | 2467 (1.6) |  |  |  |
| **7** | 5957 (3.4) | 388 (6.0) | 558 (4.4) | 5011 (3.2) |  |  |  |
| **8** | 16337 (9.4) | 910 (14.1) | 1407 (11.0) | 14020 (9.0) |  |  |  |
| **9** | 104590 (60.0) | 3567 (55.1) | 7715 (60.4) | 93308 (60.2) |  |  |  |
| **10** | 41621 (23.9) | 1149 (17.8) | 2510 (19.7) | 37962 (24.5) |  |  |  |
| **pH of the umbilical artery (mean (SD))** | 7.23 (0.23) | 7.24 (0.17) | 7.23 (0.16) | 7.23 (0.24) | 0.012 |  | 27,30 |
| **pH of the umbilical vein (mean (SD))** | 7.12 (1.16) | 7.15 (1.04) | 7.21 (0.81) | 7.11 (1.19) | <0.001 |  | 70.3 |

Table 1. Baseline characteristics of overall population (overall), women without hypertension during pregnancy (no hypertension), women with hypertensive pregnancy without pre-eclampsia (other hypertensive pregnancy) and women with pre-eclampsia (pre-eclampsia). BMI (body mass index), kg (kilogram), cm (centimeter), m (metre), min (minute), IQR (interquartile range), OGTT (oral glucose tolerance test), g (gram), SD (standard deviation), C-section (caesarean section), ICD-10 (International Classification of Diseases 10^th^ revision), F-diagnosis (mental and behavioural disorders classified in ICD-10 to F00 – F99), nonnorm (the variable is non-normally distributed)
