# Supplementary material for: External causes are leading causes of death in women of reproductive age: a registry study on maternal perinatal health, hypertensive pregnancy disorders and mortality in Finland
Source: J Epidemiol Community Health. 2025 Apr 23;79(9):e223438. doi: 10.1136/jech-2024-223438 (PMC12418550; doi:10.1136/jech-2024-223438)
Supplement: online supplemental table 2 [file jech-79-9-s003.docx]

| \|  \|  \|  \| **n** \| **deathsrate** \| **lower** \| **upper** \| \| --- \| --- \| --- \| --- \| --- \| --- \| --- \| \| **All causes of death** \| **total** \|  \| **1287** \|  \|  \|  \| \| **Preventable** \| **total** \|  \| **484** \|  \|  \|  \| \| **Violent** \| **total** \|  \| **231** \|  \|  \|  \| \|  \| Suicide \| 50 (Suicide (X60-X84, Y870)) \| 196 \| 5.42 \| 4.69 \| 6.23 \| \|  \| Assault \| 51 (Assault (X85-Y09, Y871)) \| 35 \| 0.97 \| 0.67 \| 1.35 \| \| **Accidents** \| **total** \|  \| **159** \|  \|  \|  \| \|  \|  \| 42 (Land traffic accidents) \| 45 \| 1.24 \| 0.91 \| 1.66 \| \|  \|  \| 49 (Other accidents and sequelae of accidents) \| 16 \| 0.44 \| 0.25 \| 0.72 \| \|  \|  \| 46 (Accidental falls (W00-W19)) \| 12 \| 0.33 \| 0.17 \| 0.58 \| \|  \|  \| 47 (Accidental drownings (W65-W74)) \| 7 \| 0.19 \| 0.08 \| 0.40 \| \|  \|  \| 48 (Accidental poisonings excluding accidental poisoning by alcohol (X40-X44, X46-X49, Y10-Y15)) \| 75 \| 2.07 \| 1.63 \| 2.60 \| \|  \|  \| Other accidents \| 4 \|  \|  \|  \| \| **Alcohol** \|  \| 41 (Alcohol-related diseases and accidental poisoning by alcohol) \| **94** \| 2.60 \| 2.10 \| 3.18 \| \| **Other** \|  \| 53 (Other external causes and sequelae of other external causes) \| **1** \|  \|  \|  \| \| **Malignancy** \| **total** \|  \| **493** \|  \|  \|  \| \|  \|  \| 13 (Malignant neoplasm of breast (C50)) \| 158 \| 4.37 \| 3.71 \| 5.10 \| \|  \|  \| 21 (Other malignant neoplasms) \| 122 \| 3.37 \| 2.80 \| 4.03 \| \|  \|  \| 20 (Malignant neoplasm of lymphoid, haematopoietic and related tissue (C81-C96)) \| 27 \| 0.75 \| 0.49 \| 1.09 \| \|  \|  \| 06 (Malignant neoplasm of stomach (C16)) \| 30 \| 0.83 \| 0.56 \| 1.18 \| \|  \|  \| 07 (Malignant neoplasm of colon (C18, C19)) \| 29 \| 0.80 \| 0.54 \| 1.15 \| \|  \|  \| 11 (Malignant neoplasm of larynx, trachea, bronchus and lung (C32-C34)) \| 21 \| 0.58 \| 0.36 \| 0.89 \| \|  \|  \| 14 (Malignant neoplasm of uterine cervix (C53)) \| 27 \| 0.75 \| 0.49 \| 1.09 \| \|  \|  \| 10 (Malignant neoplasm of pancreas (C25)) \| 18 \| 0.50 \| 0.29 \| 0.79 \| \|  \|  \| 16 (Malignant neoplasm of ovary (C56)) \| 22 \| 0.61 \| 0.38 \| 0.92 \| \|  \|  \| 12 (Malignant melanoma of skin (C43)) \| 13 \| 0.36 \| 0.19 \| 0.61 \| \|  \|  \| 08 (Malignant neoplasm of rectum, anus and anal canal (C20-C21)) \| 6 \| 0.17 \| 0.06 \| 0.36 \| \|  \|  \| 09 (Primary malignant neoplasm of liver and intrahepatic bile ducts (C22)) \| 14 \| 0.39 \| 0.21 \| 0.65 \| \|  \|  \| 04 (Malignant neoplasms of lip, oral cavity and pharynx (C00-C14)) \| 6 \| 0.17 \| 0.06 \| 0.36 \| \| **Cardiovas-cular disease** \| **total** \|  \| **126** \|  \|  \|  \| \|  \|  \| 29 (Cerebrovascular diseases (I60-I69)) \| 46 \| 1.27 \| 0.93 \| 1.70 \| \|  \|  \| 28 (Other heart diseases excluding rheumatic and alcohol-related (I30-I425, I427-I52)) \| 46 \| 1.27 \| 0.93 \| 1.70 \| \|  \|  \| 30 (Other diseases of the circulatory system (I00-I15, I26-I28, I70-I99)) \| 28 \| 0.77 \| 0.51 \| 1.12 \| \|  \|  \| 27 (Ischaemic heart diseases (I20-I25)) \| 6 \| 0.17 \| 0.06 \| 0.36 \| \| **Other** \| **total** \|  \| **184** \|  \|  \|  \| \|  \|  \| 26 (Other diseases of the nervous system and sense organs excluding alcohol-related) \| 25 \| 0.70 \| 0.45 \| 1.02 \| \|  \|  \| 39 (Other diseases excluding alcohol-related) \| 30 \| 0.83 \| 0.56 \| 1.18 \| \|  \|  \| 23 (Diabetes mellitus (E10-E14)) \| 23 \| 0.64 \| 0.40 \| 0.95 \| \|  \|  \| 40 (Ill-defined and unknown causes of mortality (R96-R99)) \| 14 \| 0.39 \| 0.21 \| 0.65 \| \|  \|  \| 52 (Event of undetermined intent (Y16-Y34, Y872)) \| 14 \| 0.39 \| 0.21 \| 0.65 \| \|  \|  \| 36 (Diseases of the digestive system excluding alcohol-related diseases) \| 12 \| 0.33 \| 0.17 \| 0.58 \| \|  \|  \| 03 (Other infectious and parasitic diseases (A00-A09, A20-B19, B25-B89, B91-B99)) \| 11 \| 0.30 \| 0.15 \| 0.54 \| \|  \|  \| 32 (Pneumonia (J12-J18, J849)) \| 9 \| 0.25 \| 0.11 \| 0.47 \| \|  \|  \| 24 (Other endocrine, nutritional and metabolic diseases (E00-E09, E15-E90)) \| 5 \| 0.14 \| 0.04 \| 0.32 \| \|  \|  \| 22 (Other neoplasms (D00-D48)) \| 6 \| 0.17 \| 0.06 \| 0.36 \| \|  \|  \| 38 (Congenital malformations (Q00-Q99)) \| 6 \| 0.17 \| 0.06 \| 0.36 \| \|  \|  \| 34 (Asthma (J45-J46)) \| 6 \| 0.17 \| 0.06 \| 0.36 \| \|  \|  \| Other not determined \| 23 \|  \|  \|  \| |
| --- | --- | --- | --- | --- | --- | --- | --- | --- | --- | --- | --- | --- | --- | --- | --- | --- | --- | --- | --- | --- | --- | --- | --- | --- | --- | --- | --- | --- | --- | --- | --- | --- | --- | --- | --- | --- | --- | --- | --- | --- | --- | --- | --- | --- | --- | --- | --- | --- | --- | --- | --- | --- | --- | --- | --- | --- | --- | --- | --- | --- | --- | --- | --- | --- | --- | --- | --- | --- | --- | --- | --- | --- | --- | --- | --- | --- | --- | --- | --- | --- | --- | --- | --- | --- | --- | --- | --- | --- | --- | --- | --- | --- | --- | --- | --- | --- | --- | --- | --- | --- | --- | --- | --- | --- | --- | --- | --- | --- | --- | --- | --- | --- | --- | --- | --- | --- | --- | --- | --- | --- | --- | --- | --- | --- | --- | --- | --- | --- | --- | --- | --- | --- | --- | --- | --- | --- | --- | --- | --- | --- | --- | --- | --- | --- | --- | --- | --- | --- | --- | --- | --- | --- | --- | --- | --- | --- | --- | --- | --- | --- | --- | --- | --- | --- | --- | --- | --- | --- | --- | --- | --- | --- | --- | --- | --- | --- | --- | --- | --- | --- | --- | --- | --- | --- | --- | --- | --- | --- | --- | --- | --- | --- | --- | --- | --- | --- | --- | --- | --- | --- | --- | --- | --- | --- | --- | --- | --- | --- | --- | --- | --- | --- | --- | --- | --- | --- | --- | --- | --- | --- | --- | --- | --- | --- | --- | --- | --- | --- | --- | --- | --- | --- | --- | --- | --- | --- | --- | --- | --- | --- | --- | --- | --- | --- | --- | --- | --- | --- | --- | --- | --- | --- | --- | --- | --- | --- | --- | --- | --- | --- | --- | --- | --- | --- | --- | --- | --- | --- | --- | --- | --- | --- | --- | --- | --- | --- | --- | --- | --- | --- | --- | --- | --- | --- | --- | --- | --- | --- | --- | --- | --- | --- | --- | --- | --- | --- | --- | --- | --- | --- | --- | --- | --- | --- | --- | --- | --- | --- | --- | --- | --- | --- | --- | --- | --- | --- | --- | --- | --- | --- | --- | --- | --- | --- | --- | --- | --- | --- | --- | --- | --- | --- | --- | --- | --- | --- |

Table 2

Mortality (per 100 000 person-years) for Statistics Finland classification causes of death from 2004 to 2019 [21]. The codes behind the causes refer to ICD-10 (International Classification of Diseases 10^th^ revision).
